# Effect of *Eclipta prostrata* on 11Beta-Hydroxysteroid Dehydrogenase in Rat Liver and Kidney

**DOI:** 10.1155/2014/651053

**Published:** 2014-04-30

**Authors:** Chenshu Xu, Binghua Wei, Xiaohua Fu, Meijuan Luo, Shidai Liu, Ruiming Li, Bin Ren, Lei Tang

**Affiliations:** ^1^Department of Pharmacy, The First Affiliated Hospital, Sun Yat-Sen University, 58 Zhongshan No. 2 Road, Guangzhou, Guangdong 510080, China; ^2^Department of Pharmacy, Guangzhou Xinhai Hospital, Guangzhou, Guangdong 510300, China

## Abstract

*Eclipta prostrata* (EP) is often prescribed in combination with glucocorticoid to treat glomerular nephritis, nephrotic syndrome, and IgA nephropathy in clinical practice of Traditional Chinese Medicine. Previous studies from our laboratory revealed that coadministration of EP significantly increased the plasma concentration of prednisolone while decreased the level of cotreated prednisone in rats. However, the underlying mechanism remains unclear. 11**β**-Hydroxysteroid dehydrogenase (11**β**-HSD) belongs to the family of oxidoreductases that catalyze the interconversion of prednisone to active prednisolone. Therefore, the current study aimed to investigate the effects of EP on the activity and expression of 11**β**-HSD in rat liver and kidney. The results showed that oral administration of EP significantly increased the activity of 11**β**-HSD I in the liver and 11**β**-HSD II in the kidney by employing the microsomal incubation system. Moreover, gene and protein expressions of 11**β**-HSD I and 11**β**-HSD II were also increased in rat liver and kidney, respectively. These results suggest that the effects of EP on 11**β**-HSD may attribute to the mechanism that administration of EP improves the efficacy and reduces adverse drug reactions of glucocorticoid in patients undergoing combinational therapy.

## 1. Introduction

Glucocorticoids are a class of steroid hormones that cause immunological and metabolic effects by binding to the glucocorticoid receptor. Prednisone is a synthetic corticosteroid commonly prescribed in the treatment of immunological diseases, for example, rheumatoid arthritis, systemic lupus erythematosus, polymyositis, and nephrotic syndrome. Long term therapy with prednisone has been shown to result in various adverse effects, such as myopathy, osteoporosis, hyperglycaemia, electrolyte abnormalities, hypertension, and truncal obesity. As an ester prodrug, prednisone can be rapidly converted to prednisolone, its active 11*β*-hydroxyl metabolite, by 11*β*-hydroxysteroid dehydrogenase I (11*β*-HSD I) [1].

11*β*-Hydroxysteroid dehydrogenase (11*β*-HSD) belongs to the family of oxidoreductases that regulates the access of glucocorticoids to the steroid receptors [2]. 11*β*-HSD catalyzes the interconversion of physiologic glucocorticoids such as cortisol to its inactive metabolite cortisone, as well as prednisone to active prednisolone. Moreover, 11 beta-HSD from mouse liver microsomes has also been demonstrated to catalyze the reductive metabolism of xenobiotic carbonyl compounds such as metyrapone, p-nitroacetophenone, and p-nitrobenzaldehyde [3]. There are two distinct 11*β*-HSD enzymes. 11*β*-HSD I is expressed at high levels in the liver, where orally administered prednisone is primarily activated and extensively converted to prednisolone, the active counterpart of prednisone through first-pass metabolism [4, 5]. 11*β*-HSD II, however, is a powerful inactivator of glucocorticoid and expressed primarily in the kidney, which is the principle site for generating inactive prednisone [6]. Regulation of tissue concentration of glucocorticoid by 11*β*-HSD has been shown to involve in several diseases, such as Cushing's syndrome and metabolic syndrome including central obesity, diabetes, insulin resistance, and so forth. A number of parameters were calculated to represent the enzyme activity of 11*β*-HSD, including measuring the AUC ratio of hydrocortisone/prednisolone to cortisone/prednisone in plasma [7, 8]. Ratio of (tetrahydrocortisol + 5*α*-tetrahydrocortisol)/tetrahydrocortisone [9] and ratio of (tetrahydrocorticosterone + 5*α*-tetrahydrocorticosterone)/11-dehydrotetrahydrocorticosterone [8] in urine were used to indirectly reflect the 11*β*-HSD activity. Furthermore, 11*β*-HSD activity could also be detected by microsomal incubation with radiolabeled 3H-corticosterone and 3H-cortisone as substrate [10].

*Eclipta prostrata* (EP), belonging to the family Asteraceae and commonly known as False Daisy, grows widely throughout tropical and subtropical areas, such as China, India, Thailand, and Brazil. A number of herbal preparations comprising EP are available for treatment of hepatic disorders, atherosclerosis, dementia, and abnormal uterine bleeding [11]. It has also been reported that EP possesses anti-inflammatory, antimicrobial, and antioxidation activities [12, 13]. In clinical practice of Traditional Chinese Medicine, EP is often prescribed in combination with glucocorticoid to treat glomerular nephritis, nephrotic syndrome, IgA nephropathy, and so forth. Administration of EP has been showed to improve the efficacy, reduce adverse drug reactions of glucocorticoid, and prevent recurrence of the diseases [14, 15]. Previous study from our laboratory showed that the C_max_ and AUC of glucocorticoid were significantly increased when Danmo Capsule was coadministered in rats. Danmo Capsule is a widely used herbal preparation in China whose major constituent includes EP. This study indicated the effect of EP on changing the pharmacokinetics of glucocorticoid [16]. Unpublished data also revealed that coadministration of EP significantly increased the plasma concentration of prednisolone with decreasing level of cotreated prednisone. However, the underlying mechanisms were currently unclear.

The major active constituents of EP include thiophene derivatives, triterpenes, flavonoids, polypeptides, polyacetylenes, steroids, and coumestans [17], some of which have been shown to inhibit members of short-chain dehydrogenase/reductase superfamily, including 20*α*-hydroxysteroid dehydrogenase (20*α*-HSD), 3*β*-hydroxysteroid dehydrogenase II (3*β*-HSD II), 17*β*-hydroxysteroid dehydrogenase (17*β*-HSD), and 11*β*-hydroxysteroid dehydrogenase II (11*β*-HSD II) [18, 19]. Therefore, EP extract may affect the clinical efficacy of glucocorticoid by influencing its* in vivo* activation/deactivation through 11*β*-HSD. The aim of the current study was to investigate the effects of EP on the activity and expression of 11*β*-HSD I and II in liver and kidney of rats orally administrated with different doses of EP. An HPLC method was developed and validated to determine the prednisone/prednisolone metabolism through 11*β*-HSD. By employing the microsomal incubation system, the effects of EP on 11*β*-HSD activity were investigated in liver and kidney microsomes isolated from rats treated with different doses of EP. Moreover, gene and protein expression of 11*β*-HSD in liver and kidney from the EP treated rats were also evaluated to reveal the effects of EP on 11*β*-HSD expression.

## 2. Materials and Methods

### 2.1. Experimental Animals

Male Sprague-Dawley rats (220–310 g) were purchased from the Medical Experimental Animal Center of Guangdong Province. The animals were kept in a room with a light/dark cycle of 12/12 h under controlled temperature (22 ± 2°C) and 55–60% relative humidity. They had free access to standard rodent chow and clean tap water. All procedures were in accordance with the Regulations of Experimental Animal Administration issued by the Ministry of Science and Technology of the People's Republic of China. The animal study protocols were approved by the Institutional Animal Care and Use Committee (IACUC) at Sun Yat-sen University, Guangzhou, China. Rats (*n* = 6 per group) were randomly divided and treated with control vehicle (CMC-Na), 7 g/kg, 14 g/kg or 28 g/kg of EP for 14 days before subsequent experiments.

### 2.2. Chemicals and Reagents

Prednisone, prednisolone, and dexamethasone (internal standard for HPLC analysis) were purchased from Sigma-Aldrich Corporation (St. Louis, MO, USA) and dissolved in acetonitrile to obtain working solution. Sodium carboxymethyl cellulose (CMC-Na) was also purchased from Sigma-Aldrich Corporation (St. Louis, MO, USA). Methanol and acetonitrile of HPLC grade were purchased from TEDIA Company Inc. (Beijing, China). All other reagents were of analytical grade when appropriate.

### 2.3. Preparation of* Eclipta prostrata* Extract

*Eclipta prostrata* (EP) was purchased from Guangzhou Zhixin Drug Co., Ltd. (Guangdong, China). The crude drug was dried and extracted by refluxing with 70% aqueous ethanol (1 : 30, w/v) for 2 h. After filtration, the residue was extracted again for additional 1 h 70% aqueous ethanol (1 : 15, w/v). The two extract solutions were combined and concentrated (10 : 1) by rotary evaporation below 80°C under reduced pressure. The concentrated solution was evaporated to dryness under reduced pressure. The extraction yield was approximately 14.8% (w/w). The EP extract was stored at 4°C until use. For administration to the animals, the EP extract was suspended in 1% aqueous CMC-Na, and the dose was calculated on the base of original herbs.

### 2.4. Development and Validation of an HPLC Method 

#### 2.4.1. Equipment and Chromatographic Conditions

The chromatographic system (Waters, Avondale, CA) consisted of a pump (Waters 600), an automatic injector (Waters 717), a UV detector (Waters 2489), and a Workstation (Empower). Chromatographic separation was achieved by using a C18 column (Nucleodur 100-5 C18 column, 5 *μ*m, 4.6 mm × 250 mm) at 40°C. The mobile phase consisted of (water (0.2% phosphoric acid)/methanol with the ratio of 55 : 45, A)-acetonitrile (B) and programmed by 100-100% (v/v) A at 0.0–15.0 min, 100–20% A at 15.0–18.0 min, 20-20% A at 18.0–23.0 min, 20–100% A at 23.0–28.0 min, and 100-100% A at 28.0–35.0 min. Detection was performed at wave length of 240 nm with a flow rate of 1 mL/min. The total running time was 35 min for each sample.

#### 2.4.2. Method Validation

The HPLC method was validated for the linearity of the calibration curve, accuracy, precision, and recovery. There was no significant interference at the expected retention times of the analytes and the IS. The analytes showed satisfactory linearity over the studied concentration ranges in rat tissues. The intra- and interbatch precision and accuracy were within recommended limits, and the extraction efficiency for the analyte was acceptable. Therefore, the HPLC method was validated and applicable for determining prednisone and prednisolone concentration.

### 2.5. Preparation of Rat Liver and Kidney Microsomes

Rats were euthanized with diethyl ether before liver and kidney were harvested and washed with ice-cold 0.9% NaCl solution. Tissues were homogenized in homogenization buffer (10 mmol/L Tris containing 0.25 mol/L sucrose and 1 mmol/L EDTA, pH 7.4) at 4°C. The homogenate was subjected to centrifugation at 16000 g for 20 min at 4°C. The supernatant fraction was subjected to centrifugation at 100000 g for 60 min at 4°C. The first microsomal pellet was washed and resuspended in wash buffer (0.1 mol/L potassium pyrophosphate containing 1 mmol/L EDTA, pH 7.4) and then reisolated by ultracentrifugation (100000 g for 60 min at 4°C). The washed microsomes were suspended in a small volume of Tris-HCl buffer containing 20% of glycerol and stored at −80°C until use.

### 2.6. Microsomal Incubation

The incubation system, with a total volume of 0.2 mL, contained the substrate (1 mg/mL, prednisone for liver and prednisolone for kidney), liver (5 *μ*g/*μ*L) or kidney (3 *μ*g/*μ*L) microsomes, 100 mM potassium phosphate buffer (pH 7.4), and NADPH for liver or NADP+ for kidney (1 mM). The linearity of metabolism was examined with respect to incubation time and microsomal concentration. The reaction was started by adding 20 *μ*L of NADPH to the 140 *μ*L of potassium phosphate buffer containing 20 *μ*L of microsomes and 20 *μ*L substrate that was preincubated for 5 min at 37°C. After incubation at 37°C for 25 min (liver) or 45 min (kidney), 600 *μ*L of methanol was added to the reaction mixture to stop the reaction. Then 10 *μ*L of dexamethasone (10 mg/mL, internal standard) and 600 *μ*L methanol were added to 200 *μ*L of the mixture, vortexed for 2 min, and centrifuged at 10800 rpm for 5 min. 10 *μ*L of the supernatant was injected into HPLC system for analysis. The production rate (pmol/mg protein/min) of metabolites was used to indicate enzyme activity.

### 2.7. RNA Isolation and Real-Time Quantitative-Polymerase Chain Reactions

Total RNA was extracted from the rat tissues using Trizol reagent and reverse-transcribed to complementary DNA (cDNA) using Prime script RT reagent kit. Equal amounts of cDNA were used in real-time quantitative-polymerase chain reactions (RT-QPCRs). All the PCR reactions were carried out using SYBR Premix Ex TaqTM kit (Takara, Kyoto, Japan) and followed manufacturer's instructions. The primer sets were shown in Table 1. Cycling conditions were 95°C for 30 s followed by 40 cycles of 95°C for 5 s, 60°C for 34 s, and 40°C for 60 s using the Light Cycler 2.0 Real-Time Detection System (Roche, Hercules, CA, USA). The fold induction was calculated as described previously [20].

### 2.8. Western Blot

Approximately 100 mg of rat tissues was homogenized in 1 mL of RIPA buffer and 10 *μ*L of phenylmethylsulfonyl fluoride (PMSF). Total protein was extracted by centrifugation at 15000 rpm for 30 min at 4°C and quantified by enhanced BCA protein assay kit. Protein expression was measured by Western blot analysis. Briefly, protein was resolved on 10% SDS-polyacrylamide gel and transferred to polyvinylidene difluoride microporous membranes (Millipore, Bedford, MA, USA) that were probed with anti-11*β* HSD I (Abcam, Cambridge, UK, dilution 1 : 800), anti-11 *β* HSD II (Abcam, Cambridge, UK, dilution 1 : 1000), or GAPDH (Santa Cruz Biotechnology Inc., Santa Cruz, CA, USA, dilution 1 : 500) antibodies. Immunodetection was performed using the ECL detection system according to the manufacturer's instructions. Quantitative densitometric analyses of Western blot images were achieved using ImageJ 1.44p software.

### 2.9. Statistical Analysis

Data are presented as the mean ± SD. Student's *t*-test was performed for statistical comparison of the results. The criterion of significance was set at *P* < 0.05, and tests were performed using SPSS version 10.0 software (SPSS Inc, Chicago, IL, USA).

## 3. Results

### 3.1. Effects of EP on Enzyme Activity of 11*β*-HSD I and 11*β*-HSD II in Rat Liver and Kidney

**Figure 1:**
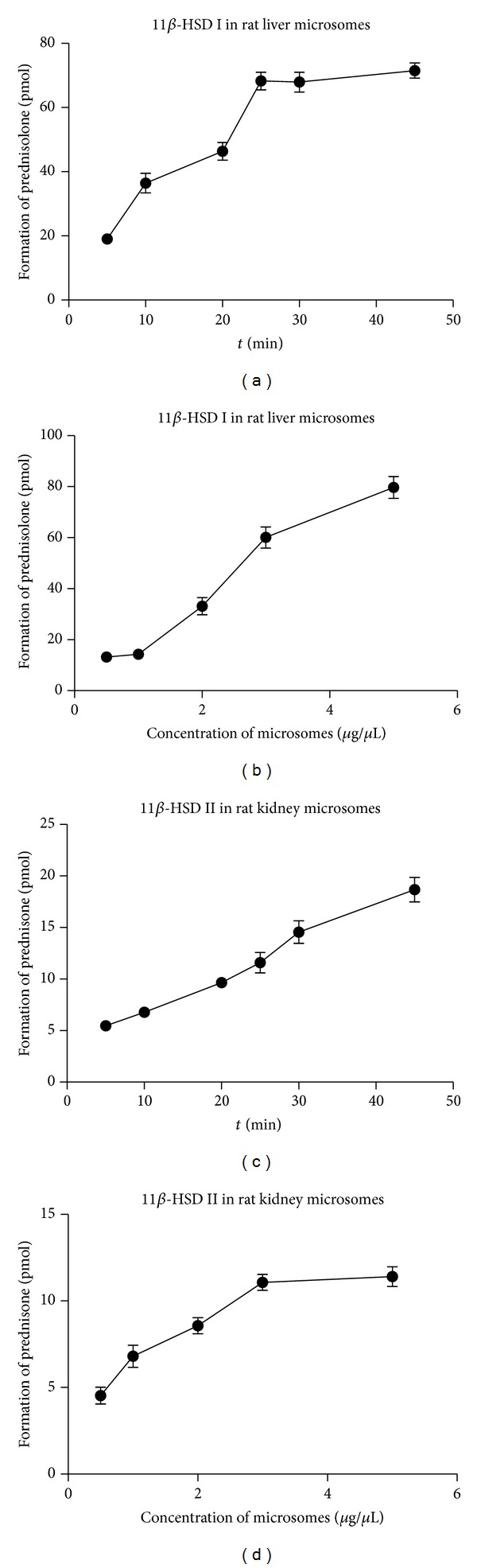
Effects of incubation time and microsome concentration on the metabolism of prednisone in rat liver and kidney microsomes. (a) The incubation system containing 5 *μ*g/*μ*L of the liver microsomes was incubated for 5, 10, 20, 25, 30, or 45 min. The analyte was injected into HPLC system to determine the effect of incubation time on the metabolism of prednisone in rat liver microsomes. (b) The incubation system containing different concentration of liver microsomes (0.5~5 *μ*g/*μ*L) was incubated for 25 min. The analyte was injected into HPLC system to determine the effect of microsome concentration on the metabolism of prednisone in rat liver microsomes. (c) The incubation system containing 3 *μ*g/*μ*L of the kidney microsomes was incubated for 5, 10, 20, 25, 30, or 45 min. The analyte was injected into HPLC system to determine the effect of incubation time on the metabolism of prednisolone in rat kidney microsomes. (d) The incubation system containing different concentration of kidney microsomes (0.5~5 *μ*g/*μ*L) was incubated for 45 min. The analyte was injected into HPLC system to determine the effect of microsome concentration on the metabolism of prednisolone in rat kidney microsomes (mean ± SD, *n* = 3).

The incubation time and microsome concentration were optimized by comparing formation of metabolite under different microsome concentrations (0.5–5 *μ*g/*μ*L) and incubation time (5–45 min). The formation of prednisolone in liver microsomes was linearly increased at the incubation time range of 5–25 min (Figure 1(a)) and over the microsome concentrations ranged from 1 to 5 *μ*g/*μ*L (Figure 1(b)). Similarly, the formation of prednisone in kidney microsomes showed a linear increase at the incubation time range of 5–45 min (Figure 1(c)) and over the microsome concentrations ranged from 1 to 5 *μ*g/*μ*L (Figure 1(d)). Therefore, the incubation condition was set as 5 *μ*g/*μ*L of liver microsomes or 3 *μ*g/*μ*L of kidney microsomes, and 25 min of incubation time for liver samples or 45 min for kidney samples.

**Figure 2:**
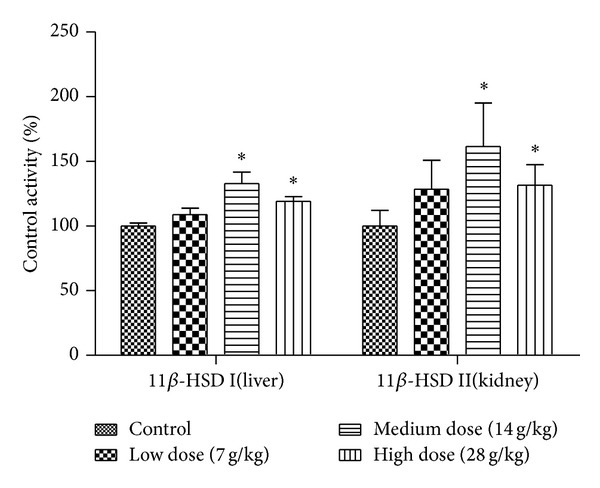
Effects of* Eclipta prostrata* on the enzyme activity of 11*β*-HSD I in liver and 11*β*-HSD II in kidney. Rats were orally administrated with vehicle (CMC-Na) or* Eclipta prostrata* (7, 14, or 28 g/kg) for 14 days. Liver and kidney microsomes were isolated and then 11*β*-HSD I and 11*β*-HSD II enzyme activities were analyzed. Data are expressed as fold change over the control group (mean ± SD, *n* = 6) *P < 0.05, **P < 0.01.

11*β*-Hydroxysteroid dehydrogenase comprises two isozymes, 11*β*-HSD I and II, which primarily locate in the microsome of liver and kidney and catalyze the interconversion of active prednisone and its inactive metabolite prednisolone [4, 5]. For the purpose of evaluating the effect of EP on the enzyme activity, rats were treated orally with vehicle (1% CMC-Na solution) or EP at the dosages of 7 g/kg, 14 g/kg, or 28 g/kg daily for 14 days. Microsomes from liver and kidney were then isolated and used in the system described above. The effects of different concentrations of EP extract on prednisone/prednisolone metabolism, which indicated the enzyme activity of 11*β*-HSD, are shown in Table 2 and Figure 2. Treatment of 14 g/kg or 28 g/kg EP significantly increased the metabolism of prednisone and prednisolone in liver and kidney, respectively. However, no difference was found in the rats treated with 7 g/kg of EP.

### 3.2. Effects of EP on Gene Expression of 11*β*-HSD I and 11*β*-HSD II in Rat Liver and Kidney

**Figure 3:**
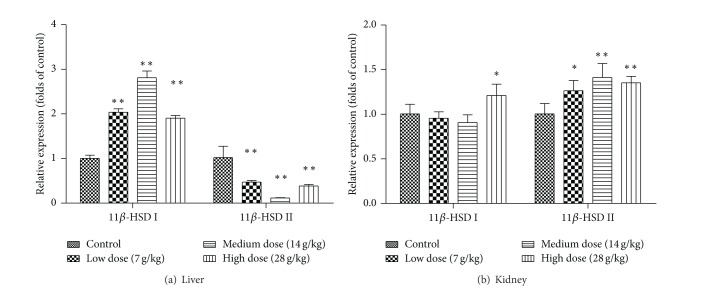
Effects of* Eclipta prostrata* on the gene expression of 11*β*-HSD I and 11*β*-HSD II in rat liver (a) and kidney (b). Rats were orally administrated with vehicle (CMC-Na) or* Eclipta prostrata *(7, 14, or 28 g/kg) for 14 days. Liver and kidney were harvested and then 11*β*-HSD I and 11*β*-HSD II mRNA levels were analyzed. Data are expressed as fold change over the control group (mean ± SD, *n* = 6) *P < 0.05, **P < 0.01.

In order to investigate the effect of EP on the gene expression of 11*β*-HSD I and 11*β*-HSD II in rat liver and kidney, 24 rats were randomly divided into 4 groups and treated orally with vehicle (1% CMC-Na solution) or EP at the dosages of 7 g/kg, 14 g/kg, or 28 g/kg daily for 14 days. On the morning of day 15, tissues were harvested, and total RNA was isolated from the liver and kidney. The gene expressions of 11*β*-HSD I and 11*β*-HSD II were analyzed by real-time quantitative PCR. Treatment of EP (7, 14, and 28 g/kg) significantly induced the gene expression of 11*β*-HSD I by 103%, 181%, and 90%, whereas those of 11*β*-HSD II were reduced by 53%, 89%, and 63% in the liver, respectively (Figure 3(a)). In contrast, the expression levels of 11*β*-HSD II in the kidney were markedly induced by different doses of EP treatments. However, only 28 g/kg of EP induced the expression of 11*β*-HSD I in the kidney (Figure 3(b)).

### 3.3. Effects of EP on Protein Expression of 11*β*-HSD I and 11*β*-HSD II in Rat Liver and Kidney

**Figure 4:**
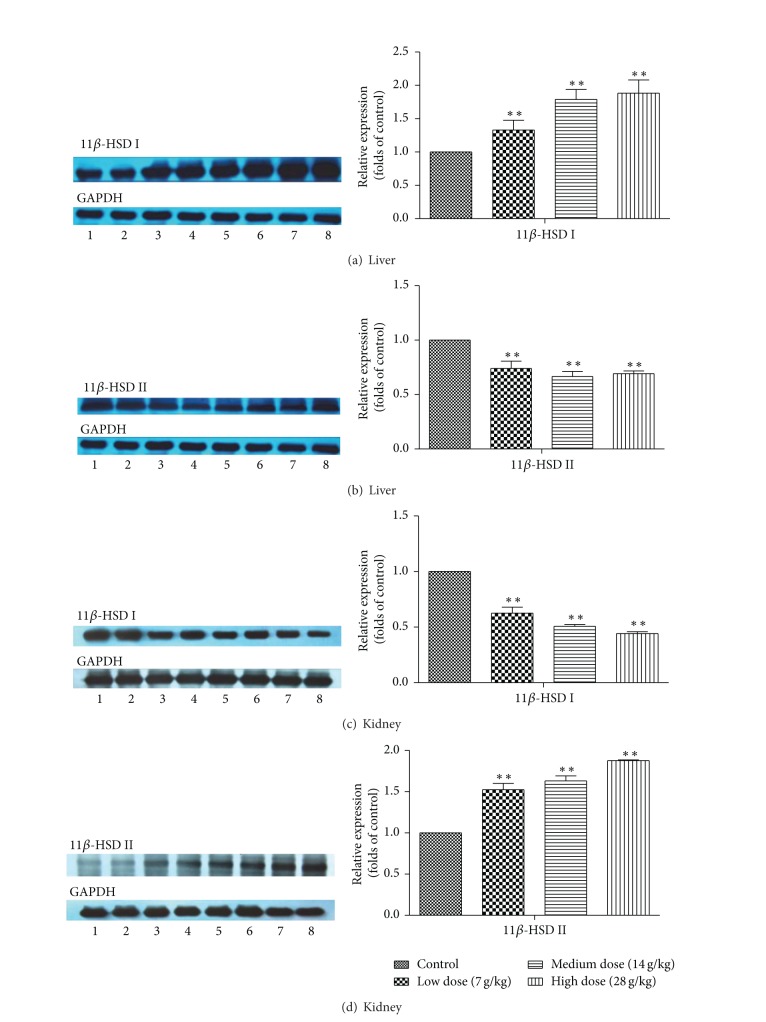
Effects of* Eclipta prostrata* on the protein expression of 11*β*-HSD I and 11*β*-HSD II in rat liver (a and b) and kidney (c and d). Rats were orally administrated with vehicle (CMC-Na) or* Eclipta prostrata* (7, 14, or 28 g/kg) for 14 days. Liver and kidney were harvested and then 11*β*-HSD I and 11*β*-HSD II protein levels were analyzed. Data are expressed as fold change over the control group (mean ± SD, *n* = 6) *P < 0.05, **P < 0.01. 1, 2: control group; 3, 4:* Eclipta prostrata *7 g/kg; 5, 6:* Eclipta prostrata* 14 g/kg; 7, 8:* Eclipta prostrata* 28 g/kg.

We next examined the relative expression levels of 11*β*-HSD I and 11*β*-HSD II protein in rat liver and kidney. Total proteins were isolated and resolved on an SDS-polyacrylamide gel for subsequent Western blot analysis. Expression of 11*β*-HSD I protein in the liver was induced by EP (7, 14, and 28 g/kg) treatment, whereas that of 11*β*-HSD II was reduced (Figures 4(a) and 4(b)). Quantitative analyses of these data showed that protein expression of 11*β*-HSD I was increased by EP (7, 14, and 28 g/kg) treatment by 33%, 78%, and 88%, whereas that of 11*β*-HSD II was reduced by 26%, 34%, and 31%, respectively. In contrast, treatment of EP (7, 14, and 28 g/kg) in the kidney revealed an opposite effect that protein expression of 11*β*-HSD I was reduced by 38%, 49%, and 56%, while that of 11*β*-HSD II was increased by 52%, 63%, and 88% when compared with vehicle treated group (Figures 4(c) and 4(d)).

## 4. Discussion

In the current study, an HPLC method was developed and validated to determine the prednisone/prednisolone metabolism through 11*β*-HSD. Several chromatographic studies have been reported to determine the concentration of prednisone/prednisolone in biological samples, including HPLC-UV, HPLC-FID, LC-MS/MS, and GC-MS [21–23], among which HPLC-UV was widely used for its relative inexpensiveness and simplicity in operation. In the present HPLC method we developed, acetonitrile water was originally employed as the mobile phase but yielded significant peak tailing for prednisone/prednisolone. Therefore, the mobile phase was optimized and phosphoric acid was added to obtain better peak shapes. Thus, a mobile phase consisted of (water (0.2% phosphoric acid)/methanol with the ratio of 55 : 45)-acetonitrile was chosen to achieve good peak shapes with proper retention time.

Microsomal incubation system is widely applied in the studies of enzyme activity and drug metabolism* in vitro*. One of the aims of our study was to examine the effect of oral administration of EP on prednisone/prednisolone metabolism through 11*β*-HSD in liver and kidney. Thus, prednisone and prednisolone were used as the substrate for evaluating the enzyme activity of 11*β*-HSD I and 11*β*-HSD II in the microsomal incubation, respectively. In order to completely reflect the enzyme activity, 100 *μ*g/mL of substrate was chosen, at the concentration of which the metabolism of substrate reached plateau. Prednisone/prednisolone is insoluble in water but is soluble in methanol which may inhibit the enzyme activity in microsomal incubation. Different concentration of methanol was compared, and 10% of methanol was chosen as solvent for substrate, at the concentration of which the substrate was dissolved without affecting the enzyme activity.

11*β*-Hydroxysteroid dehydrogenase (11*β*-HSD) catalyzes the conversion of inert 11 keto-products (cortisone) to active cortisol, or vice versa, thus regulating the local concentration of glucocorticoids in liver and kidney [24]. 11*β*-HSD I is expressed extensively in the liver, where prednisone is activated and converted to prednisolone through 11*β*-HSD I to exert its pharmacological effect [4]. Therefore, prednisolone should be given to patients with impaired liver function to avoid insufficient efficacy because of reduced enzyme activity. In contrast, 11*β*-HSD II is expressed mainly in the kidney and is responsible for the inactivation of cortisol [6]. Deficiency of 11*β*-HSD II may result in accumulation of cortisol, which activates the mineralocorticoid receptor in the distal renal tubule and increases side effects, such as water-sodium retention, hypertension, and hypopotassemia. In the current study, oral administration of EP significantly increased the activity of liver 11*β*-HSD I and kidney 11*β*-HSD II in microsomal incubation system, which partially explain the effect that coadministration of EP and glucocorticoid improve the efficacy and reduce adverse drug reactions of glucocorticoid.

In order to determine whether the effect of EP on 11*β*-HSD activity was mediated through influencing the 11*β*-HSD expression in liver and kidney, gene and protein levels of 11*β*-HSD I and II from rats orally treated with EP were evaluated. The trends of elevation in expression of 11*β*-HSD I in liver and 11*β*-HSD II in kidney were consistent with their activities. However, the expression of 11*β*-HSD II in liver and 11*β*-HSD I in kidney showed an opposite effect with the exception that the mRNA expression of 11*β*-HSD I in kidney was significantly increased when 28 g/kg of EP was treated. The effect of EP on protein expression revealed a dose-dependent manner, while the dosage of EP at 14 g/kg exerted the biggest effect on mRNA expression and enzyme activity. Posttranscriptional events and alteration in protein stability may attribute to the discrepancy.

Prednisone has been identified as a substrate of P-glycoprotein (P-gp), a well-characterized ABC-transporter which transports a wide variety of substrates across extra- and intracellular membranes [25]. Methylprednisolone (MP) can also be taken up in the intestinal epithelial cells by a carrier-mediated transport mechanism, in which the absorptive and secretory clearance of MP increased and decreased, respectively, in the presence of P-gp inhibitors [26]. The involvement of CYP3A4, one of the major cytochromes P450s responsible for the biotransformation of nearly 60% of all clinically prescribed drugs in human, has also been revealed in the 6 beta-hydroxylation of prednisone [27]. The effects of EP on the above drug metabolizing enzymes and transporter proteins are currently unclear. It has been identified that rifampicin, the prototypical ligand for pregnane x receptor (PXR), reduces effectiveness and bioavailability of prednisolone [28]. As the nuclear receptor PXR has been well established as the master regulator of genes encoding multiple drug metabolizing enzymes and transporter proteins including CYP3A4 and P-gp, the effects of EP on the PXR-mediated regulation of its target genes may also contribute to the alteration in glucocorticoid levels* in vivo*.

To further elucidate the underlying mechanisms of how cotreatment with EP improves glucocorticoid efficacy, extra studies should be conducted. Glucocorticoids act through the glucocorticoid receptor (GR), another member of the nuclear receptor superfamily. As a transcription factor, GR binds to glucocorticoid response elements (GRE) and regulates the gene transcription. GR can also modulate the activity of other transcription factors [29, 30]. Therefore, it is of particular interest to determine whether EP exerts its effects through regulating the GR function.

## 5. Conclusion

In summary, oral administration of EP significantly increased the activity and expression of 11*β*-HSD I in the liver and 11*β*-HSD II in the kidney, which may attribute to the effect that administration of EP improves the efficacy and reduces adverse drug reactions of glucocorticoid in patients undergoing combinational therapy.

## Figures and Tables

**Table 1:** Primer sequences used for real-time polymerase chain reaction.

Gene	5′-3′ sequence	PCR condition
11*β*-HDS I		Stage 1: Predenaturation (30 s at 95°C) 1 Cycle
Forward Reverse	TGACCAAGGTCAACGTGTCCA ATGATCTCCAGGGCGCATTC	
11*β*-HDS II		
Forward Reverse	GACCTTAGCCCCGTTGTAGATG GGCAGGTAGTGGTGGATGAAA	Stage 2: PCR reaction (5 s at 95°C) (20 s at 60°C) (60 s at 40°C) 40 Cycle
*β*-Actin		
Forward Reverse	GGAGATTACTGCCCTGGCTCCTA GACTCATCGTACTCCTGCTTGCTG	

**Table 2:** Effects of *Eclipta prostrata* (7 g/kg, 14 g/kg, and 28 g/kg) on the metabolism of prednisone and prednisolone (1 mg/mL) in rat liver and kidney microsomes (*n* = 6).

Treatment	Activities of enzymes (pmol/min per mg protein)	
	11*β*-HSD I (liver microsomes)	11*β*-HSD II (kidney microsomes)
Control	383.8 ± 9.0	22.6 ± 2.7
*Eclipta prostrata* (g/kg)		
7	418.2 ± 18.9	29.0 ± 5.1
14	510.1 ± 33.9*	36.5 ± 7.6*
28	457.2 ± 14.1*	29.7 ± 3.6*

*P < 0.05 versus control. Data are mean ± SD.
